# The Court of Arbitration for Sport's Decision in IOC v. Valieva: An Example of Arbitral Activism or Necessary Arbitral Activity?

**DOI:** 10.3389/fspor.2022.924916

**Published:** 2022-06-27

**Authors:** Marcus Mazzucco

**Affiliations:** Faculty of Kinesiology and Physical Education, University of Toronto, Toronto, ON, Canada

**Keywords:** sports arbitration, CAS, judicial activism, accountability, athletes' rights, doping, WADA, democracy

## Introduction

On February 14, 2022, a panel of the Court of Arbitration for Sport (CAS) announced its decision permitting 15-year-old Russian figure skater Kamila Valieva to compete at the 2022 Olympic Games, despite rules in the World Anti-Doping Code (WADC) that mandated her provisional suspension due to a positive drug test (International Olympic Committee et al., 2022). The CAS panel's decision was met with swift opposition from Olympic movement stakeholders, including athletes and national Olympic committees, who raised concerns about the effectiveness of the anti-doping system to ensure clean sport (Elliott, 2022; Frisk, 2022; Park, 2022). However, the sharpest criticism of the CAS panel's decision came from the World Anti-Doping Agency (WADA), which effectively accused the panel of judicial activism by rewriting the WADC in a manner that was neither intended by its drafters nor proposed by any of the stakeholders consulted on the 2021 version of the WADC (World Anti-Doping Agency, 2022).

WADA's comments are reminiscent of the criticisms that courts face when they invalidate laws to protect individual rights. More specifically, courts are accused of engaging in judicial activism by exceeding their role to interpret and apply laws and by encroaching on the law-making role of democratically elected legislative branches that represent the “will of the people” (Court of Appeal for Ontario, 1999; Barak, 2001; Policy Options, 2003; Supreme Court of Canada, 2004). However, these concerns are often misguided and based on a lack of understanding of a court's jurisdiction and its institutional role within a national legal system (Court of Appeal for Ontario, 1999; Barak, 2001; Policy Options, 2003; Supreme Court of Canada, 2004).

This paper will explore the CAS panel's decision in the Valieva case by situating it within the legal authority of arbitrators and the necessary institutional role that CAS ought to serve in the international sport system. This institutional role ensures that international sport organizations are held legally accountable for violating athletes' fundamental rights, and that athletes are able to participate in the evolution of sport rules in a manner not otherwise permitted due to the undemocratic and unrepresentative nature of governance structures in international sport organizations.

## The Valieva Case

On December 25, 2021, Valieva was selected for an in-competition doping control test at the 2022 Russian National Figure Skating Championships (International Olympic Committee et al., 2022). The urine sample she provided revealed the presence of the prohibited substance trimetazidine; however, this was not determined until February 7, 2022, after Valieva had competed in the women's singles free skate component of the team event at the 2022 Olympic Games (International Olympic Committee et al., 2022). Trimetazidine is classified as a non-specified substance that is prohibited at all times in WADA's Prohibited List and its presence in an athlete's urine sample triggers a mandatory provisional suspension under the WADC, unless the athlete can show that the presence of the prohibited substance was likely the result of a contaminated product (World Anti-Doping Agency, 2021a). Initially, Valieva was provisionally suspended by the Russian Anti-Doping Agency (RUSADA) but she challenged the suspension^1^ and it was lifted by RUSADA's disciplinary panel following a hearing.^2^ On February 11th and 12th, the International Olympic Committee (IOC), WADA, and the International Skating Union filed separate appeals to challenge the disciplinary panel's decision before the CAS *Ad Hoc* Division operating at the Olympic Games. Valieva, RUSADA, and the Russian Olympic Committee were named as respondents in the appeals (International Olympic Committee et al., 2022).

The CAS panel dismissed the appeals and decided not to reinstate Valieva's provisional suspension, thereby allowing her to compete at the 2022 Olympic Games (International Olympic Committee et al., 2022). The panel found that Valieva, due to her young age, was a “protected person” within the meaning of the WADC and that there appeared to be a gap or lacuna in the WADC rules regarding the imposition of mandatory provisional suspensions against protected persons (International Olympic Committee et al., 2022). The panel observed that the WADC includes special rules for protected persons that provide them with lesser sanctions, lower evidentiary burdens, and greater privacy rights compared to non-protected persons “in light of their age and inexperience, and their diminished responsibility for rule violations” (International Olympic Committee et al., 2022, p. 35). Yet, the panel found that the imposition of a mandatory provisional suspension on a protected person could lead to instances where the provisional suspension is more severe than the ultimate sanction that would be imposed following a full hearing (e.g., a reprimand and no period of ineligibility) where the protected person establishes that their level of fault or negligence was not significant in relation to the rule violation (International Olympic Committee et al., 2022). In the panel's view, this outcome was likely to occur due to a protected person's youth, immaturity, and lack of legal capacity—all of which are factors used to assess fault or negligence under the WADC (International Olympic Committee et al., 2022). In addition, this disparity between the severity of provisional and ultimate sanctions did not exist for *older* athletes who are not protected persons, and thus resulted in the different and harsher treatment of protected persons^3^ (International Olympic Committee et al., 2022). The panel reasoned that such outcomes indicated a gap in the rules for mandatory provisional suspensions that could not have been intended by the drafters of the WADC (International Olympic Committee et al., 2022). The panel held that it had the authority to fill this gap to avoid an overly harsh or inconsistent outcome based on the fundamental principle of proportionality^4^ upon which all legal systems are based (International Olympic Committee et al., 2022). Accordingly, the panel concluded that protected persons, such as Valieva, who are accused of anti-doping rule violations should not be subject to the WADC rules regarding mandatory provisional suspensions^5^ (International Olympic Committee et al., 2022).

In reaching the above conclusion, the panel was careful to note that its decision was an exercise in interpreting the WADC and not an example of “rewriting rules or making policies that are better made by sporting bodies exercising proper governance” (International Olympic Committee et al., 2022, p. 36). The panel further emphasized that “it does not see itself as a policymaker or rulemaker, but it is properly called upon, as are courts around the world, to interpret rules and how they work” (International Olympic Committee et al., 2022, p. 36).

## CAS's Scope of Authority

CAS's intervention in the Valieva case is defensible when one considers the scope of the arbitration panel's authority. Arbitrators obtain their authority from the arbitration agreement entered into between disputing parties and other sources of arbitral powers (Bjorklund, 2018). In the sport context, an arbitration agreement usually consists of an arbitration clause contained in an entry form for a sport competition or a sport organization's regulations. If the arbitration agreement designates a particular institution to administer the arbitral process (e.g., CAS), then the agreement will typically state that the arbitration will be conducted in accordance with the procedural rules of the institution.

In the case of Valieva, the arbitration agreement consisted of the entry form that Valieva signed to compete in the Olympic Games and the arbitration clause in rule 61.2 of the IOC's Olympic Charter^6^ (International Olympic Committee et al., 2022). The procedural rules for the CAS *Ad Hoc* Division authorize a panel to decide a dispute pursuant to the Olympic Charter, applicable regulations, as well as the general principles of law and rules of law, the application of which the panel deems appropriate^7^ (Court of Arbitration for Sport, 2021a). A CAS panel's authority to decide a dispute based on laws external to sport rules is integral to its judicial role. Sport rules are not autonomous texts that are immune from external legal principles and norms. For example, CAS panels have noted that the rules of WADA and the IOC must comply with Swiss law, as they are both private law entities subject to Swiss law (Mariano Puerta, 2006; International Olympic Committee, 2016). In Valieva's case, the CAS panel followed the procedural rules of the *Ad Hoc* Division and attempted to apply the WADC to decide the dispute. It was only after identifying a gap in the WADC that the panel resorted to applying the fundamental legal principle of proportionality found in Swiss law and other systems of law to interpret the WADC in a manner that avoided violating this principle.

A CAS panel's reliance on external legal rules and norms also arises from its inherent powers that are a necessary consequence of its judicial nature and its duty to ensure the administration of justice (Bjorklund, 2018), both of which are facilitated by the Swiss *Private International Law Act* (Federal Act on Private International Law, 1987). This Act allows CAS decisions to be reviewed by the Swiss Federal Tribunal for their compatibility with Swiss public policy.^8^ As a result, a CAS panel has an obligation to consider the fundamental legal rights that comprise Swiss public policy, such as those enshrined in human rights law, to avoid having its decisions overturned by the Swiss Federal Tribunal (International Olympic Committee, 2016; Lindholm, 2019).

## CAS's Necessary Institutional Role

The arbitration panel's reliance on the fundamental principle of proportionality to interpret the WADC in the Valieva case was not only authorized but necessary in light of CAS's institutional role in the international sport system. It is well-established that international sport organizations are neither representative of nor politically accountable to athletes, even though athletes are the ultimate subjects of their regulatory authority (Houlihan, 2004; Geeraert et al., 2013; Freeburn, 2018). As Houlihan (2004) notes, athletes are often demoted to the sidelines of anti-doping policy development based on the false assumption that their interests are the same as those of sport organizations, and that sport organizations will act in the best interests of athletes. Unlike other international sport organizations, WADA is not an association of members. Instead, it is a private law foundation that is led by a 38-member foundation board, with a 14-member executive board and several standing committees (World Anti-Doping Agency, 2021a). Historically, this governance structure has not adequately represented athletes or given athletes a fair share of decision-making power (Houlihan, 2004; Geeraert et al., 2013). It was only in 2021 that WADA committed to increase the number of seats for athlete representation on its foundation board (from four seats to six), on its executive board (from one seat to two), and on its standing committees (at least one seat), and to ensure that its standing athlete committee was comprised of athletes selected by external athlete associations, and not the President of WADA (World Anti-Doping Agency, 2021a). Although this increase in athlete representation is a good step forward, it does not make WADA democratically or politically accountable to athletes.

In the context of the international anti-doping system, the absence of political accountability in relation to athletes has several implications for CAS's institutional role. First, without political accountability, athletes must resort to legal means, such as arbitration, to hold international sport organizations accountable (Lang, 2008). As Lang notes, judicial bodies belong among the most important institutions by which an individual may “flip the coin” in its subordinate relationship with a governing entity and hold it accountable for non-fulfillment of the rule of law (Lang, 2008). Second, arbitration provides athletes with opportunities to participate in the evolution of sport rules—albeit in a retrospective manner, as opposed to a proactive manner (Geeraert et al., 2013)—and thereby assume a role that they would not otherwise have due to their lack of representation and decision-making power within the governance structures of sport organizations. In other words, arbitration becomes a means for athletes to advocate for change in a given policy area and challenge rules that infringe on their rights and interests. Third, because anti-doping rules are not made by democratically elected officials, there should be no reluctance on the part of arbitrators to critically review the substance of the rules based on a fear of interfering with the “will of the people.” As Freeburn (2021) notes, the democratic legitimacy of governments is what entitles governments to rely upon public interest justifications to defend laws that violate individual rights. However, because international sport organizations lack legitimacy associated with governmental power, the basis upon which they should be permitted to justify violations of human rights using public policy considerations should be questioned by arbitrators (Freeburn, 2021).

## Conclusion

The arbitration panel's decision in the Valieva case is a good example of CAS's authority and necessary institutional role to scrutinize the substance of sport rules through the application of external legal rules. Rather than be accused of engaging in arbitral activism and overstepping its role as a judicial body, CAS should be encouraged to exercise these lawful and necessary arbitral activities as it is increasingly being called upon by athletes and other stakeholders to adjudicate human rights disputes in sport. If CAS fails to exercise this authority and assume this role, then it risks weakening its institutional legitimacy and being relegated to a mere enforcer of sport rules made by undemocratic and unrepresentative international sport organizations that often disregard the rights and interests of athletes (Foster, 2006; Mazzucco and Findlay, 2010; Freeburn, 2018, 2021). Such an outcome would leave athletes without an effective legal mechanism to hold sport organizations to account and without a strategic means to shape the evolution of sport policies.

## Author Contributions

The author confirms being the sole contributor of this work and has approved it for publication.

## Conflict of Interest

The author declares that the research was conducted in the absence of any commercial or financial relationships that could be construed as a potential conflict of interest.

## Publisher's Note

All claims expressed in this article are solely those of the authors and do not necessarily represent those of their affiliated organizations, or those of the publisher, the editors and the reviewers. Any product that may be evaluated in this article, or claim that may be made by its manufacturer, is not guaranteed or endorsed by the publisher.

## Footnotes

^1^Valieva's challenge to the provisional suspension was necessary so that she could compete in subsequent events pending the outcome of a full hearing on the merits of her alleged anti-doping rule violation, including the women's singles event at the Olympic Games scheduled to begin on February 15, 2022.

^2^The disciplinary panel lifted the provisional suspension after concluding that the presence of the prohibited substance in Valieva's urine sample was likely due to a contaminated product and therefore did not trigger a mandatory provisional suspension under anti-doping rules (International Olympic Committee et al., 2022). The disciplinary panel also held that a provisional suspension would be a disproportionate penalty in the circumstances (International Olympic Committee et al., 2022).

^3^Older athletes whose anti-doping rule violations involve a contaminated product, or a specified substance, and no significant fault or negligence can receive a reprimand and no period of ineligibility as an ultimate sanction, similar to a protected person who demonstrates no significant fault or negligence. However, older athletes in these circumstances can also avoid a mandatory provisional suspension, whereas a protected person cannot (unless the protected person's violation involves a contaminated product or a specified substance) (World Anti-Doping Agency, 2021b).

^4^The principle of proportionality provides that a sanction must be proportionate to a rule violation.

^5^The panel held that, instead of a mandatory provisional suspension, protected persons should be subject to an optional provisional suspension in accordance with article 7.4.2 of the WADC. Article 7.4.2 permits anti-doping organizations to adopt their own rules for imposing a provisional suspension where the suspension is not mandated by the WADC. However, the panel concluded that, under the facts and circumstances, a provisional suspension should not be imposed on Valieva. Although the panel did not specify which facts and circumstances it was referring to in this part of the decision, several facts noted elsewhere in the decision appear to be relevant. First, at this stage in the case, there was no proven or admitted anti-doping rule violation; rather, there was an asserted violation based on an adverse analytical finding relating to Valieva's “A” sample. The amount of the prohibited substance found in Valieva's “A” sample was below the technical limit of detection and Valieva intended to request an analysis of her “B” sample. Second, although the likelihood of Valieva's success on the merits of the case could not be evaluated in depth at this stage, she appeared to have some factual basis to argue that the violation was due to no significant fault or negligence, which, if proven on a balance of probabilities at a full hearing, could result in little to no period of ineligibility. Finally, if a provisional suspension was imposed, and the subsequent full hearing concluded that no period of ineligibility should be served or that the period of ineligibility should be less than that which was already served under a provisional suspension, then Valieva's inability to compete at the Olympic Games (due to a provisional suspension) would cause her irreparable harm (International Olympic Committee et al., 2022).

^6^Rule 61.2 of the Olympic Charter provides that any dispute arising on the occasion of, or in connection with the Olympic Games, shall be submitted exclusively to CAS, in accordance with CAS's procedural rules (International Olympic Committee, 2021).

^7^The procedural rules of CAS's other divisions (i.e., Ordinary, Appeals, and Anti-Doping Divisions) similarly allow panels to apply the laws of a particular jurisdiction, on a primary or subsidiary basis (Court of Arbitration for Sport, 2021b,c). Generally speaking, the applicable state law, and whether it will be applied to resolve a dispute on a primary basis or on a basis that is subsidiary to applicable sport rules, will depend on the agreement of the parties.

^8^More specifically, the PILA applies where at least one party to the CAS arbitration is domiciled outside of Switzerland (article 176(1), PILA). Where all parties are domiciled in Switzerland, the arbitration is domestic (not international) and is subject to the *Swiss Civil Procedure Code* (CPC), which also permits challenges to arbitration decisions on narrow grounds (see article 393, CPC). However, because the vast majority of CAS arbitrations are international, this paper focuses on the PILA.
